# Loss of Nuclear/DNA Integrity in Mouse Epididymal Spermatozoa after Short-Term Exposure to Low Doses of Dibutyl Phthalate or Bisphenol AF and Its Mitigation by Oral Antioxidant Supplementation

**DOI:** 10.3390/antiox12051046

**Published:** 2023-05-05

**Authors:** Elisa Hug, Pauline Villeneuve, Stephanie Bravard, Areski Chorfa, Christelle Damon-Soubeyrand, Stephen G. Somkuti, Aron Moazamian, R. John Aitken, Parviz Gharagozloo, Joël R. Drevet, Fabrice Saez

**Affiliations:** 1 GReD Institute, CNRS UMR6293-Université Clermont Auvergne, Faculté de Médecine, CRBC, 28 Place Henri Dunant, 63001 Clermont-Ferrand, France; 2 Sincera Reproductive Medicine, Fort Washington, PA 19034, USA; 3 CellOxess LLC, Ewing, NJ 08540, USA; 4 School of Environmental and Life Sciences, Priority Research Centre for Reproductive Sciences, The University of Newcastle, Callaghan, Newcastle 2308, Australia

**Keywords:** endocrine disruptors, environmental pollutants, oxidative stress, DNA damage, spermatozoa, male fertility

## Abstract

Routine exposure to chemicals omnipresent in the environment, particularly the so-called endocrine-disrupting chemicals (EDCs), has been associated with decreased sperm quality and increased anomalies in testis. The decline in semen quality and testicular abnormalities have been attributed to the disruption of endocrine signaling as well as oxidative stress. The present study set out to examine the effect of short-term exposure of two common EDCs widely used in the plastic industry: Dibutyl Phthalate (DBP) and Bisphenol AF (BPAF). Our research objective was to focus on the post-testicular compartment of the epididymis, where spermatozoa acquire their functional capacity and are stored. The data obtained indicated no significant effect for either chemicals on sperm viability, motility or acrosome integrity. Neither of the EDCs had a noticeable effect on the structures of the testis and epididymis. However, substantial impact on the integrity of the sperm nucleus and DNA structure was evidenced by a significant increase in nuclear decondensation and DNA base oxidation. The damage observed was postulated to arise from the pro-oxidant properties of the EDCs generating excess of reactive oxygen species (ROS) and triggering a state of oxidative stress. This hypothesis was confirmed when the observed damage was largely blocked by co-administering EDCs with an evidenced-based antioxidant formulation.

## 1. Introduction

Earlier but also large recent meta-analyses of semen parameters point to a 50% fall in human sperm counts since 1973 [1,2,3,4,5,6]. Equally alarmingly, a large percentage of men considering IVF treatment show abnormal levels of sperm DNA damage with roughly 1 in 10 having high levels of DNA fragmentation, potentially exposing the female partner to higher rates of miscarriage and deleterious genetic changes in offspring [7]. Modern lifestyles and poor nutrition, as well as a whole variety of environmental factors are the likely causes for the deterioration of semen quality including sperm DNA integrity. Environmental chemicals, particularly endocrine-disrupting chemicals (EDCs), are major contributors to the progressive decline in semen quality and sperm DNA integrity [8,9]. The EDCs are ubiquitous contaminants in the human body, wildlife, and the environment [10,11,12]. They interfere with many aspects of mammalian hormonal homeostasis, leading to unbalanced situations which disturb physiology, including reproductive function. The effect of EDCs on male reproductive function has been analyzed with a logical major focus on how it affects spermatogenesis. Amongst EDCs, phthalates, and bisphenols, two families of very common environmental pollutants have been extensively studied [13].

Phthalates, esters of phthalic acid, are a family of common environmental chemicals present in a wide variety of items including toys, cosmetics, pharmaceuticals and construction materials. Exposure over time can be toxic to humans either by ingestion, contact and breathing [14]. Identified as anti-androgenic [15], phthalates are recognized as EDCs due to their male reprotoxic properties as documented by several studies in animal models [16,17,18,19]. The endocrine-disruptive properties of phthalates and their pro-oxidant activity lead to ROS-mediated testicular apoptosis and autophagy [19]. In more details, in adult rats, 15 days of exposure to high doses (200, 400 or 600 mg/kg/day) of di-butyl phthalate (DBP) can decrease serum follicle stimulating hormone and testosterone levels, due to the direct effect of DBP on Sertoli and Leydig cells [17]. High doses (2 g/kg/day) for 14 days of di-2ethylhexyl phthalate (DEHP) can also trigger spermatocyte apoptosis by significantly decreasing the expression levels of HSP70 and Bcl-2 and increasing the expression level of cytochrome c, caspase-3 expression and activity, Bax expression and FasL content in the testes [18]. In addition, rats exposed to DHEP (at 250 and 500 mg/kg/day for 35 days) exhibit blood-testis barrier (BTB) destruction due to excessive ROS-mediated autophagy [19]. Phthalates are indeed known to generate oxidative stress, as rat exposure to high doses (200, 400 or 600 mg/kg/day) of DBP for 15 days showed a decrease in the activities of testicular antioxidant enzymes, including superoxide dismutase, catalase and glutathione reductase [17].

Bisphenols (BPs) are also widely used in plastic industry to manufacture polycarbonate and epoxy resins that make up most of our food and beverage containers [20]. These compounds can leach into our food from the protective internal epoxy resin coatings of canned foods and from consumer products such as polycarbonate tableware, food storage containers, water bottles and baby bottles [21,22]. Bisphenol A, was among the first in its class to enter widespread use since the 1960s. It is now well-established that BPA contributes to many pathologies including cardiovascular, obesity, thyroid dysfunction, cancer and infertility [23]. The evidence for infertility is supported when urine BPA levels was linked to poor semen quality [24] and perturbation of spermatogenesis both in adult and young animal models [25,26]. The detrimental BPA effects on mammalian spermatogenesis were shown to be mediated by its endocrine-disrupting action and by its oxidizing potential [27,28]. More specifically, in rats, BPA was shown to increase serum LH levels and to decrease serum testosterone levels by inhibiting Leydig and Sertoli cell functions [25]. Long (60 days) exposure to BPA (2 to 200 µg/kg/day) impaired spermatogenesis by disrupting meiotic progression and inducing testicular cell apoptosis [26]. Due to its reprotoxic concerns, the use of BPA in food industry was limited or even banned in many countries. However, several cousin analogues or structural mimetics were introduced by industry as supposedly safer replacements [29]. These include bisphenol S (BPS), bisphenol F (BPF) and more recently, bisphenol AF (BPAF) a fluorinated analog of BPA [29]. Among these, BPAF is the least studied congener. Early studies suggest adverse impact to the testis development, increasing levels of ROS generation, disruption of the blood-testis-barrier (BTB) and a decrease in sperm motility [30]. Although both phthalates and bisphenol compounds are associated with reprotoxicity in men, in particular, details concerning their effects on sperm DNA structural integrity are still lacking. Additionally, almost all studies have focused on using high doses of these agents over long exposure times, raising questions as to the validity of the experimental protocols [17,18,19,25,26,31,32,33,34].

While dealing with ingested molecules which end up in the systemic compartment, from which the testis is rather well preserved, we considered it important to monitor what was happening in a more blood-permeant male accessory organ, such as the epididymis [35]. In the present study using animal models, we have evaluated the effect of short time exposures with low doses of DBP or BPAF on epididymal spermatozoa structure and function. The epididymis was chosen as tissue of choice as it confers to testis-produced spermatozoon most of its functional abilities (i.e., motility and ability to fertilize an oocyte). Our most recent investigations have shown that in contrast to the testis, the epididymis is highly irrigated [35], thus allowing pollutants to come into contact with transiting sperm cells in an oxidizing environment. Since redox homeostasis for epididymal sperm maturation is critical [36,37], we examined the integrity of the epididymis structure and possible impact to epididymal sperm cells on exposure to both phthalates and BPs as they can potentially induce a state of oxidative stress. To confirm our hypothesis, we co-administered a validated antioxidant supplement trademarked Fertilix^®^ [38] to examine if any damage resulting from exposure to EDCs can be ameliorated or even blocked. 

## 2. Materials & Methods

### 2.1. Animal Ethics Statement

Wild-type C57bl/6 male mice (bred in an in-house animal facility), aged 8 to 10 months, were used throughout the study. Animals were housed in polypropylene cages (1 to 6 animals/cage following French regulatory procedures on animal experimentation) under a 12/12-h, light/dark cycle, and acclimated in an environmentally controlled room (room temperature 24 ± 2 °C, relative humidity 40–50%, frequent ventilation). Animals were fed a basal diet (Global-diet, 2016S, Harlan, Gannat, France) *ad libitum* and had free access to water (each day water consumption was visually monitored by looking for classic signs of dehydration in the animals in order to assess treatment intake). This study was approved by the Comité Régional d’Ethique pour l’Expérimentation Animale (CEMEA-Auvergne) and the French Ministry of Higher Education and Research (APAFIS #33605-2021101917156568 v4).

### 2.2. Exposure to Pollutants

Mice were divided into four treatment groups *per* pollutant (5 to 8 animals/group randomly assigned), as follows: (1) a control group, (2) a group exposed to the pollutant at 50 mg/kg/day (Bisphenol AF or Dibutyl Phthalate, Sigma-Aldrich, Saint Louis, MO, USA), (3) a group exposed to an oral antioxidant supplementation (Fertlix^®^, CellOxess, Ewing, NJ, USA), and (4) a group co-exposed to the pollutant and oral antioxidant supplementation simultaneously. The pollutants and antioxidant supplementations were administered via dilution in drinking water and the mice were exposed for 14 days. At the end of the exposure, the mice were anesthetized with isoflurane and a cardiac puncture was performed to collect blood plasma prior to euthanasia via cervical dislocation for tissue collection. Preliminary studies (not shown) were conducted upstream comparing several low doses (2, 10 and 50 mg/kg/day) of pollutants. We chose the 50 mg/kg/day dose for the present study because of the appearance of significant effects, specifically with this dose, on sperm DNA oxidation. We also tested the effect of both pollutants, under the same exposure conditions, on CD1 mice. However, as these mice were less sensitive to sperm DNA oxidation than C57/BL6 mice, we decided to use the more sensitive mice for the final study.

### 2.3. Spermatozoa Recovery 

Epididymides were removed and the caput and cauda regions divided before transferring to a glass dish containing 500 µL of M2 medium (Sigma-Merck, Saint-Quentin-Fallavier, France) for sperm retrieval. To recover the spermatozoa, epididymides tails were squeezed with forceps and then punctured several times with a 26 G needle. After 10 min of incubation at 37 °C to allow sperm dispersion, these preparations were washed with 500 µL of M2 medium to obtain 1 mL of final total volume. Sperm count was determined using a Malassez hemocytometer.

### 2.4. Tissue Histology 

One caput epididymis and one testis *per* animal were fixed in 4% paraformaldehyde (Euromedex, Souffelweyersheim, France) for 24 h and 48 h, respectively. Tissues were then dehydrated in an increasing ethanol gradient and placed in Histoclear (HS200; National Diagnostics, Atlanta, GA, USA) for 2 h before paraffin embedding. Five µm-thick sections were made using a microtome (HM340E; Thermo Fisher Scientific, Illkirch, France). Tissue sections were then deparaffinized by passage through Histoclear and progressively rehydrated in a decreasing ethanol gradient. Masson’s Trichrome staining was performed using an automated deparaffinization and staining machine (HMS 70, Microm) and the slides were finally mounted with Cytoseal (Thermo Fisher Scientific, Illkirch, France). The photomicrographs presented for epididymal histology are focused on the caput segment 1/segment 2 boundary. This is because this region of the mouse epididymis caput has notable characteristics (in terms of, among others, tubule size/shape, presence of stereocilia and spermatozoa in the lumen of the tubules, cell distribution, etc.). Moreover, segment 1 of the mouse caput is highly irrigated by blood vessels, which makes it the most exposed segment to any circulating molecule.

### 2.5. Total Protein Extraction

Epididymal tissues (caput and corpus) were homogenized in 300 µL of lysis buffer (25 mM Hepes, 0.4 M NaCl, 1.5 mM MgCl_2_, 0.2 mM EDTA, NP-40 1%) and kept on ice for 20 min. The homogenates were centrifuged at 16,000× *g* for 10 min at 4 °C. The supernatant was retained, and total protein concentration was quantified using the Braford Protein Assay Kit II (Bio-Rad, Hercules, CA, USA). Absorbance was measured by the Multiskan Go microplate spectrophotometer (Thermo Fischer Scientific, Waltham, MA, USA). Differing concentrations of bovine serum albumin (BSA) were used as calibration standards to determine protein concentrations. The optical densities of the samples were determined at 595 nm.

### 2.6. Plasma Total Antioxidant Capacity (TAC)

Following anesthetization, blood samples were collected via cardiac puncture and plasma was prepared and cryopreserved (−20 °C) until processed. Plasma samples (40 µL each) were subjected to an electrochemical Total Antioxidant Capacity assessment using the e-BQC lab^TM^ device, as recommended by the manufacturer (Bioquochem, Llanera, Spain). The redox potential measurements obtained are expressed in charge-units micro-Coulombs (µC). 

### 2.7. 4-Hydoxynonenal Analysis (4-HNE)

Slot blots were performed as described in Diniz et al., 2022 [39]. Briefly, each protein extract was diluted to a concentration of 0.05 µg/µL with phosphate buffer saline (PBS) and transferred to a polyvinylidene difluoride (PVDF) membrane. The membrane was previously activated for 1 min in methanol, rinsed for 5 min in sterile water, and for a final 15 min in PBS. The slot blot was performed using a Slot Blot Manifold (Hoefer Scientific Instruments, San Francisco, CA, USA). Membranes were blocked for 60 min with 5% skim milk in Tris-buffer containing 0.05% Tween 20 (TBS-T) and then incubated overnight with a goat anti-4-Hydroxynonenal primary antibody (dilution 1:5000; Merck Millipore, reference AB5605, Darmstadt, Germany). 4-HNE visualization was achieved via a secondary antibody (rabbit anti-goat IgG-HRP; at 0.1 µg/mL; BI2403) and revealed with WesternBright™ ECL (Advansta, CA, USA) using the Chemidoc MP imaging system (Bio-Rad, Hercules, CA, USA). The density of each band was obtained with Image J33 software.

### 2.8. Standard Sperm Parameters (Mobility, Viability and Acrosome Integrity) Assessment

The Sperm Class Analyser platform (SCA, Microptic, Barcelona, Spain) was used to evaluate several standard sperm parameters. Sperm motility analyses were performed on sperm suspensions that were diluted by one-third with M2 medium and placed in a pre-warmed 20 µm deep slide (IMV, L’Aigle, France). The SCA recording and motility parameters were as follows: frame rate: 50 frames per second (FPS); particle area: 11–40 µm^2^; VLC cutoff values: fast > 25 < medium > 15 < slow > 10; progressivity: >80% of STR; connectivity: 30 pixels; and VAP points: 5 pixels. At least 500 spermatozoa were analyzed *per* mouse. Sperm viability analysis was performed using the FluoVit kit (Microptic) which distinguishes viable spermatozoa (blue) from dead spermatozoa stained (red). At least 300 spermatozoa *per* smear were counted. Acrosome integrity was performed using the FluoAcro kit (Microptic).

### 2.9. Sperm Nuclear and DNA Integrity Tests

Sperm nuclear condensation was assessed by Toluidine blue (TB) staining [40], where a smear of 100,000 sperm *per* sample was placed on a glass slide and then stained with 1% TB in McIlvain buffer (200 mM Na_2_HPO_4_, 100 mM citric acid, pH 3.5) for 17 min at room temperature. The slides were dehydrated in ethanol and mounted with Cytoseal 60 medium. At least 300 spermatozoa *per* smear were counted. 

Sperm DNA fragmentation was assessed by the terminal deoxynucleotidyl transferase dUTP nick end labeling (TUNEL) method via the “in situ cell death detection” kit (Roche Molecular Biochemicals, Mannheim, Germany) and following modifications published by Lloyd (2020) [41]. Sperm DNA was counterstained using Hoechst 33342 (at 0.001 mg/mL). A positive control was performed by incubating sperm with 0.02% H_2_O_2_ for one hour at room temperature in the dark. The percentage of sperm with fragmented DNA was then determined by flow cytometry using the ATTUNE NxT machine (Life Technologies, Carlsbad, CA, USA). 

The level of oxidation of the sperm nucleus was assessed by measurement of 8-hydroxy-2-deoxyguanosine (8-OHdG) residue content via immunofluorescence as reported previously [42] (antibody clone 15A3 at 0.1 mg/mL, ab183393, Abcam). Sperm DNA was counterstained with Hoechst 33342 (0.001 mg/mL) for 5 min. The sperm suspension was then spread on a glass slide, mounted with Mowiol (Euromedex, Souffelweyersheim, France) and at least 300 sperm *per* smear were counted using the SCA platform (Microptic, Barcelona, Spain).

### 2.10. Statistics 

Non-parametric Mann-Whitney tests were performed with GraphPad Prism 5.02 software to determine statistically significant differences between samples. For slot blot analysis, a one-sample *t*-test was performed with GraphPad Prism 5.02 software to determine statistically significant differences between the exposed mice and the control group. *p* values ≤ 0.05 were considered significant.

## 3. Results

### 3.1. Short-Term, Low-Dose Exposure to DBP or BPAF Did Not Affect the Histological Characteristics of the Testis or Epididymis

**Figure 1:**
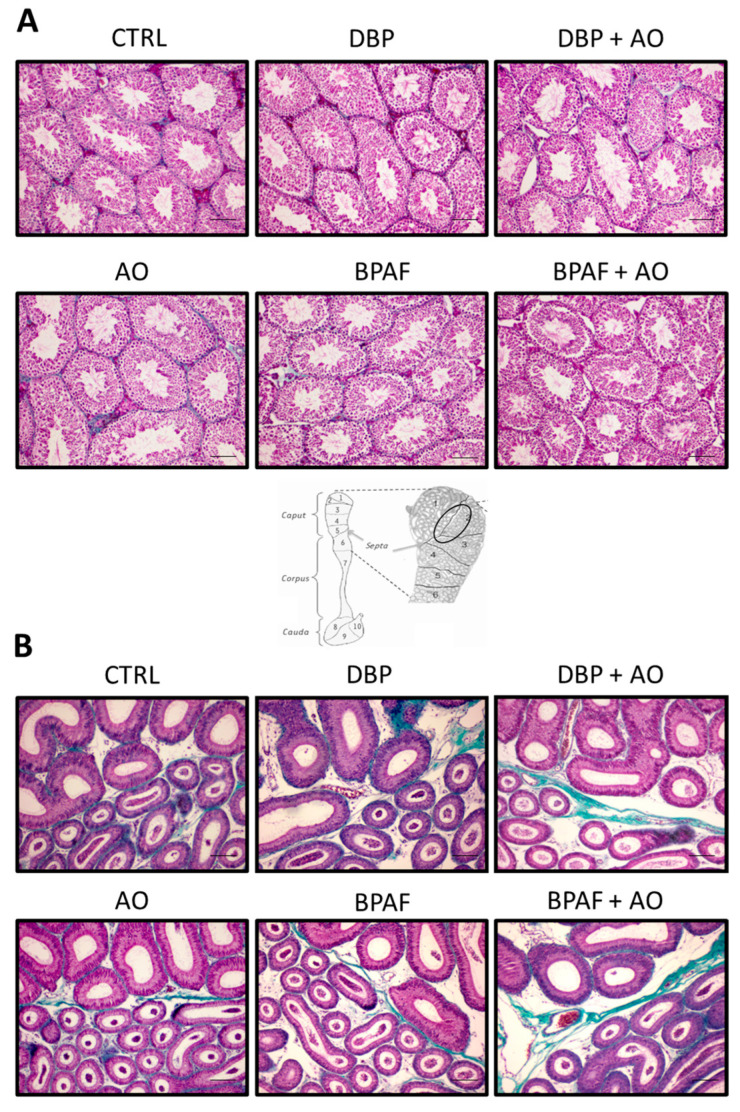
Short-term, low-dose exposure to DBP or BPAF has no impact on the histology of the testis and epididymis. Photomicrographs of the testis (**A**) and epididymis at the caput ½ segments junction (**B**) after staining with Masson’s trichrome on paraffin sections (5 µm). Selected photomicrographs are representative of at least 6 animals per group. CTRL represents control sections and AO indicates sections from antioxidant-supplemented animals.

The structures of the testes and epididymides were analysed with Masson’s Trichrome staining. Exposures to both EDCs had no effect on the macroscopic and microscopic structures of the testis and epididymis. Seminiferous tubules were well circular, not vacuolated and had sperm in their lumen (Figure 1A). Exposure of male mice to antioxidant supplementation, alone or with pollutants, also did not cause any macroscopic testicular histological modification. The histology of the epididymis caput (segment 1/segment 2 territory taken as a reference, see Figure 1) remained normal under all conditions (Figure 1B). As expected, and similar to the control sections, the caput epididymal epithelium was regular and thicker in segment 1 than in segment 2, and spermatozoa were visible in the lumen of segment 2 in all groups. The interstitium and septa (green in Figure 1B) showed no sign of fibrosis. 

### 3.2. Short-Term, Low-Dose Exposure to DBP or BPAF Triggered Oxidative Stress

**Figure 2:**
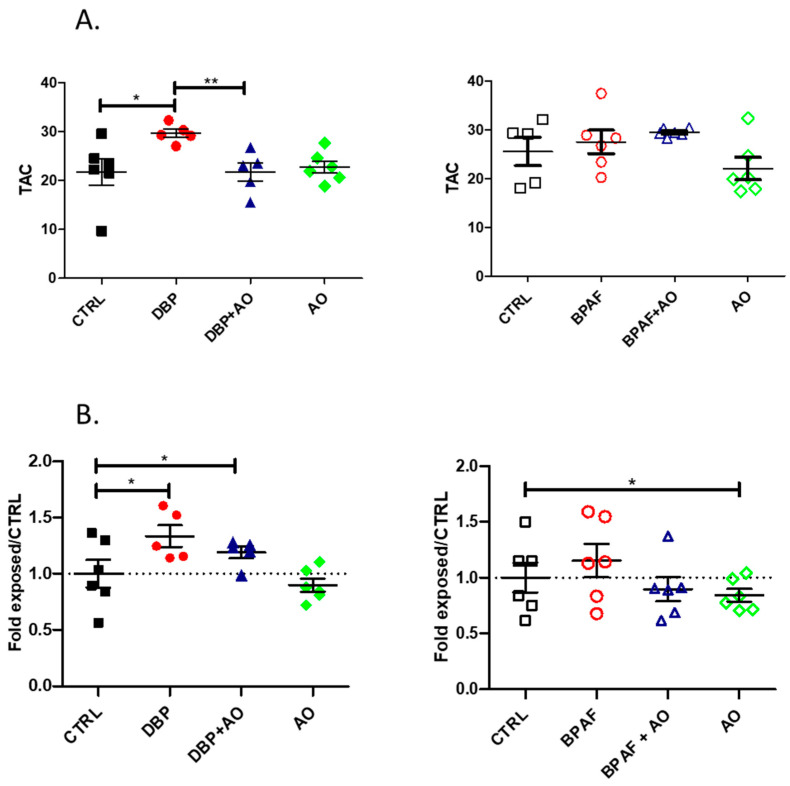
Short-term, low-dose exposure to DBP but not BPAF triggers systemic and epididymal oxidative stress. (**A**) vertical scatter plots showing plasma total antioxidant capacity (TAC) levels of different groups of animals, assessed by electrochemistry. The TAC redox potential measurements obtained are expressed in charge-units micro-Coulombs (µC). Mann-Whitney statistical evaluation was performed and the p values presented are as follows: * *p* < 0.05; ** *p* < 0.01 (**B**) vertical scatter plots presenting the 4-HNE content in protein extracts of caput epididymis tissue, assessed by slot-immunoblot and densitometry analysis. Data are expressed as fold change relative to control. Statistically significant differences (*p* < 0.05) are indicated by * compared with control. Results are represented as mean ± SEM. The number of mice *per* group varied between 5 and 6. DBP, dibutyl phthalate; BPAF, bisphenol AF; 4-HNE, 4-hydroxynonenal; CTRL, control; AO, antioxidant.

To determine whether orally administered pollutants, at a low dose and for a short duration, induced a state of oxidative stress, two parameters were measured. First, the total antioxidant capacity (TAC) of the plasma of the different animal groups was evaluated to assess systemic oxidative stress (Figure 2A). Second, the 4-HNE content of caput epididymis tissue extract was measured (Figure 2B) to assess local oxidative stress. We observed that DBP exposure was able to significantly induce a systemic antioxidant response as evidenced by increased plasma TAC levels (Figure 2A, left). DBP was also able to induce significant levels of epididymal oxidative stress confirmed by higher levels of 4-HNE adducts in caput extracts (Figure 2B, left). DBP was more potent than BPAF in this regard, as only trends toward increased systemic levels of TAC and 4-HNE content in the caput epididymis were recorded (Figure 2A,B, right panels). Co-administration with Fertilix^®^ significantly reduced the impact of DBP on plasma TAC and 4-HNE levels in the caput epididymis (Figure 2A,B, left).

### 3.3. Short-Term, Low-Dose Exposure to DBP or BPAF Did Not Affect Classical Sperm Parameters

Although long exposure times at high doses of EDCs are well documented as altering semen parameters and sperm fertilizing competence, we questioned whether low-dose exposure for a short time had any effect. As reported in Table 1 and Table 2, only BPAF exposure significantly reduced cauda epididymal sperm count (SC). All the other parameters monitored, including spermatozoa viability (SV), total mobility (SM), progressive motility (SPM) and acrosome integrity (SA), remained unchanged (Table 1 and Table 2). Oral co-administration of Fertilix^®^ with BPAF did not restore cauda sperm count (Table 2), whereas it significantly improved the progressive motility of the DBP-treated animal spermatozoa. The results suggest that the significant negative impact of BPAF on sperm count is not mediated through oxidative stress and other mechanisms are likely involved which will be discussed thereafter. 

### 3.4. Short-Term, Low-Dose Exposure to DBP or BPAF Significantly Altered Sperm DNA Integrity

**Figure 3:**
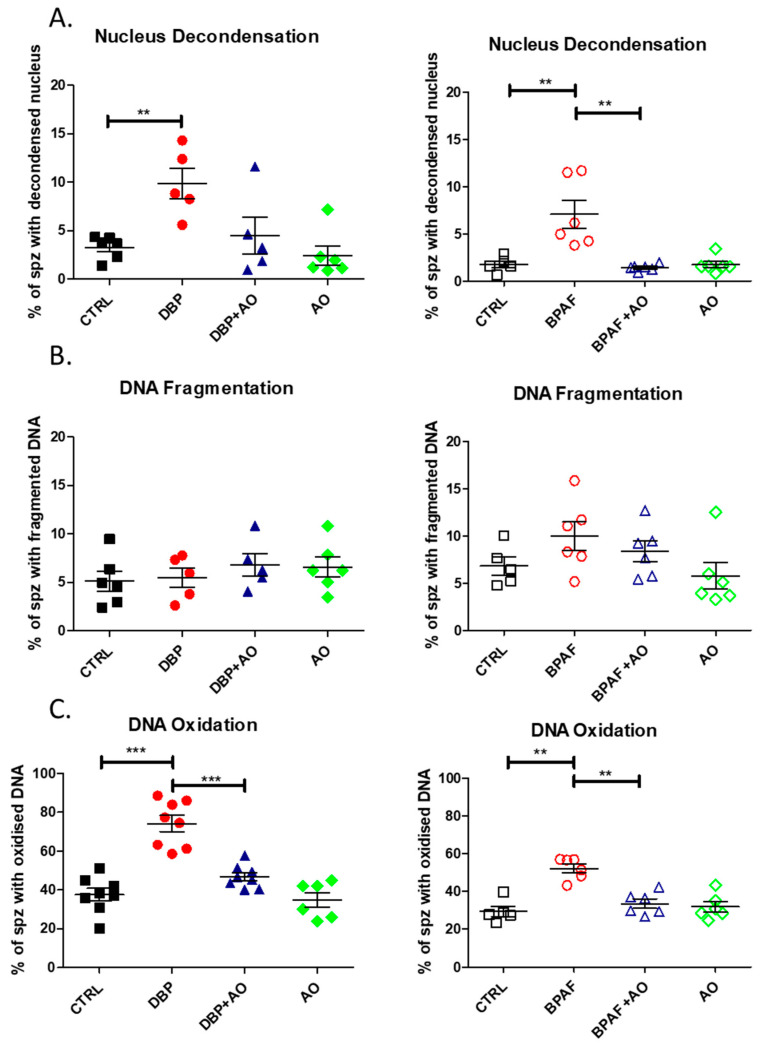
Nuclear/DNA integrity analysis of sperm collected from the caudal epididymis after exposure to DBP or BPAF. (**A**) vertical scatter plots showing the percentage of spermatozoa with a decondensed nucleus, estimated by toluidine blue staining. (**B**) vertical scatter plots showing the percentage of spermatozoa with DNA fragmentation via TUNEL assay and flow cytometry. (**C**) vertical scatter plots showing the percentage of spermatozoa with DNA base oxidation by 8-OHdG immunofluorescence. Exposure to DBP is shown on the left graphs, while exposure to BPAF is shown on the right graphs. Mann-Whitney statistical evaluation was performed and the p values presented are as follows: ** <0.01; *** <0.001. The number of mice *per* group varied between 5 and 8. CTRL indicates unexposed control animal and AO refers to antioxidant supplemented animals.

Since oxidative stress adversely impacts sperm nuclear and DNA integrity, we examined three distinct but interconnected parameters to assess the quality of the sperm paternal genetic material. Sperm nuclear condensation (Figure 3A) was significantly affected in the animal groups to which the EDCs were administered: BPAF (*p* < 0.01) or DBP (*p* < 0.01). In both cases, co-administration with Fertilix^®^ supplementation blocked the damage and maintained sperm nuclear condensation levels observed in the control groups. Sperm DNA fragmentation was not significantly changed in any of the animal groups, although BPAF exposure showed a tendency to increase it (Figure 3B). However, sperm DNA oxidation was significantly increased on exposure to BPAF (*p* < 0.01) and DBP (*p* < 0.001). Co-administration of Fertilix^®^ completely prevented the oxidation of sperm DNA induced by both EDCs.

## 4. Discussion

The significant deleterious effects of EDCs on normal spermatogenesis and male fertility are well documented by numerous studies [17,18,25,43,44]. However, little or no information is available on their impact on spermatozoa during post-testicular maturation, particularly during their transit through the epididymal compartment. Moreover, most of the available data reflect the results of studies with high doses of EDCs [17,18,19] and long exposure times [26,30], to cover at least one spermatogenic cycle. To our knowledge, milder conditions such as low dose exposure of EDCs over a short period of time has not been investigated. In addition, it is not known if the negative impact of EDCs on semen parameters [17,30,32,45] is testicular, post-testicular or both. To address these questions, male mice were fed with low doses of two EDCs (50 mg/kg/day), which are widely used in the plastic industry (DBP and BPAF, a structural analogue of BPA recently introduced as a replacement), for two weeks to cover epididymal sperm maturation. Anticipating the epididymal disruptive action of the EDCs on spermatozoa would be mediated by their pro-oxidant activity, and an evidence-based commercial antioxidant formulation (Fertilix^®^) was co-administered to mitigate possible damage to spermatozoa. In contrast to previous reports that long-term exposure of mice to high doses of EDCs caused testicular atrophy [46], no histological defects in either the testis or the epididymis was noticeable using our experimental conditions. Consistent with previous reports on the pro-oxidative action of phthalates and BPs [27,31], the exposure conditions in our experiments (low dose with a short duration) were sufficient to generate a state of oxidative stress. This was made evident, especially for the DBP exposure, by a rise in the plasma Total Antioxidant Capacity (TAC), an early cellular response to oxidative stress. In the epididymal compartment, 4-hydroxynonenal (4-HNE) content, a biomarker of lipid peroxidation, was significantly elevated in epididymal caput protein extracts [47]. DBP appeared to be a more powerful oxidant than BPAF in our experiments, inducing higher epididymal caput 4-HNE levels. Surprisingly BPAF exposure only induced a mild rise of epididymal caput 4-HNE and plasma TAC levels. This could be due to interindividual differences or because the C57bl/6 strain is more sensitive to phthalates than to BPs, as it is known that there are certain specificities related to the genetic background of the mice used [48]. However, in experimental setups published by another group, BPAF was reported to be a particularly good inducer of oxidative damage, even at low concentrations [49]. 

The results from our experiments suggest little or no change to classical sperm parameters, including motility (total and progressive), viability, morphology and acrosome integrity of caudal epididymal spermatozoa. The only change observed was a significant reduction in caudal epididymal sperm counts but this was specific to BPAF exposure. The mechanism for the reduction in sperm counts could be due to the potent cell cycle arrest action of BPAF [50]. It has been reported earlier that BPAF exerts strong toxicity (in a caspase-dependent pro-apoptotic manner) towards spermatogonial cells and pre-adipocyte stem cells, even at low concentrations and a short duration of exposure [51,52]. Although sperm structures appeared to be intact, we observed a significant impact on sperm nuclear condensation and sperm DNA oxidation, two co-dependent parameters [53,54]. Both exposures caused an increase in the number of spermatozoa with a decondensed nucleus as well as spermatozoa with DNA base oxidation. These alterations in the sperm nuclear compartment are typical of post-testicular environment under oxidative stress [36,37,53,54,55] at least for DBP exposure. However, the exposures did not result in increased sperm DNA fragmentation, confirming the presence of a mild systemic and epididymal oxidative stress. This observation is fully congruent with the phenotype we reported earlier for the mouse *Gpx5^−/−^* transgenic strain, where a mild epididymis oxidative stress led to high levels of sperm DNA oxidation but not DNA fragmentation [55]. The results are interesting and noteworthy since without the assessment of sperm DNA oxidation, we would have assumed that the EDCs used in the experiments caused no alteration in sperm nuclear/DNA integrity at low doses and for short-term exposures. This is almost always the case in Assisted Reproductive Techniques (ART) where at best patients are only evaluated for sperm DNA fragmentation to determine sperm DNA quality. The measurement of sperm DNA oxidation is never considered even though it has been linked with abnormal embryonic development, miscarriages and perinatal mortality in animal models [55]. Sperm DNA oxidation does not affect fertilization rate [55] but it does increase the burden on the oocyte DNA base-excision repair pathway to replace oxidized bases on the male nucleus. Incomplete removal of DNA-oxidized bases in the male nucleus by the oocyte may promote the propagation of transversion mutations in the developing embryo [56], potentially leading to pregnancy loss or the transmission of deleterious mutations to the future generations. The fact that oral antioxidant supplementation effectively mitigated the nuclear damage observed in spermatozoa of the exposed mice confirms that the mild epididymal oxidative environment triggered by the environmental pollutants, such as EDCs, was mainly responsible for the observed effect. The antioxidant protection offered by Fertilix^®^ against EDCs is in complete agreement with our previous results where Fertilix^®^ supplementation prevented oxidative alteration observed in the *Gpx5^−/−^* sperm nucleus [38]. 

## 5. Conclusions

The results from our experiments confirm that even a brief exposure to low doses of the so-called endocrine-disrupting chemicals such as DBP and BPAF omnipresent in our environment can impact post-testicular sperm integrity, critically its most important component, the paternal genetic material. In summary, chemical exposure can result in significant deterioration of sperm DNA integrity with little or no apparent effect on standard sperm parameters. It is important to keep in mind that almost always, the standard WHO-recommended semen parameters, but not sperm DNA integrity, are measured to assess semen quality in men seeking reproductive assistance. Therefore, it would be clinically relevant to routinely assess sperm DNA integrity, including DNA fragmentation, DNA oxidation and nuclear condensation, to better assess the genetic integrity of a given sperm sample. This could have great diagnostic and prognostic value for the success of ART, including the quality of life of subsequent generations.

It is however reassuring to confirm here that the oxidative damage sustained by spermatozoa can be mitigated by a properly formulated antioxidant supplementation. If the results observed in mice translate to man, evaluation of sperm DNA integrity, both DNA oxidation and fragmentation, and subsequent preventive treatment by evidenced-based antioxidants should become part of the standard of care at IVF centres prior to treatment by ART.

## 6. Study Limitations

It would have been interesting to have plasma and/or tissue levels of the pollutants and their metabolites to complete our analysis. In addition, an analysis of the reproductive performance of males exposed to the pollutants could have told us whether the damage caused to sperm cells would have had an impact on male fertility.

## Figures and Tables

**Table 1:** Conventional spermatozoa parameters of control mice and mice exposed to DBP with or without antioxidant (AO).

Exposure	SC (M/mL)	SV	SM	SPM	SA
Control	16.0 ± 2.9	38.6 ± 2.3	21.2 ± 4.1	15.6 ± 3.9	42.3 ± 1.6
DBP	18.6 ± 0.9	38.7 ± 2.4	22.3 ± 2.5	16.8 ± 2.4	51.1 ± 4.4
DBP + AO	18.4 ± 2.0	38.6 ± 2.0	30.5 ± 3.6	26.5 ± 3.8 (a)	57.1 ± 5.2
AO	16.9 ± 1.8	41.7 ± 1.9	22.8 ± 1.8	16.7 ± 1.6	49.4 ± 2.2

Values are presented as mean ± SEM. DBP, Dibutyl phthalate; AO, antioxidant (Fertilix^®^); SC, Sperm Count; SV, Sperm Viability; SM, Sperm total Motility; SPM, Sperm Progressive Motility; SA, Sperm acrosome integrity. (a) *p* < 0.05 in comparison with DBP exposed group. The number of mice *per* group varied between 6 and 8.

**Table 2:** Conventional spermatozoa parameters of control mice and mice exposed to BPAF with or without antioxidant (AO).

Exposure	SC (M/mL)	SV	SM	SPM	SA
Control	19.4 ± 2.2	48.6 ± 2.4	31.6 ± 2.8	25.2 ± 1.5	49.6 ± 8.1
BPAF	11.7 ± 1.4 (a)	46.8 ± 1.0	26.4 ± 2.6	21.2 ± 2.4	46.0 ± 4.7
BPAF + AO	12.0 ± 1.1 (a)	46.5 ± 2.6	28.2 ± 4.4	25.3 ± 4.5	40.7 ± 3.0
AO	19.7 ± 1.5	55.0 ± 2.7	34.6 ± 4.2	23.0 ± 5.3	59.6 ± 5.1

Values are presented as mean ± SEM. BPAF bisphenol AF; AO, antioxidant (Fertilix^®^); SC, Sperm Count; SV, Sperm Viability; SM, Sperm total Motility; SPM, Sperm Progressive Motility; SA, Sperm acrosome integrity. (a) *p* < 0.05 in comparison with the control group, (The number of mice *per* group varied between 5 and 6.

## Data Availability

Data is contained within the article.

## References

[1] Levine H., Jørgensen N., Martino-Andrade A., Mendolia J., Weksler-Derri D., Jolles M., Pinotti R., Swan S.H. (2023). Temporal trends in sperm count: A systematic review and meta-regression analysis of samples collected globally in the 20th and 21st centuries. Hum. Reprod. Update.

[2] Levine H., Jørgensen N., Martino-Andrade A., Mendolia J., Weksler-Derri D., Mindilis I., Pinotti R., Swan S.H. (2017). Temporal trends in sperm count: A systematic review and meta-regression analysis. Hum. Reprod. Update.

[3] Jørgensen N., Lamb D.J., Levine H., Pastuszak A.W., Sigalos J.T., Swan S.H., Eisenberg A.W. (2021). Are worldwide sperm counts declining?. Fertil. Steril..

[4] Carlsen E., Giwercman A., Keiding N., Skakkebaek N.E. (1992). Evidence for decreasing quality of semen during past 50 years. BMJ.

[5] Sengupta P., Dutta S., Krajewska-Kulak E. (2017). The Disappearing Sperms: Analysis of Reports Published Between 1980 and 2015. Am. J. Mens Health.

[6] Swan S.H., Elkin E.P. (1999). Declining semen quality: Can the past inform the present?. BioEssays.

[7] Aitken R.J. (2022). Role of sperm DNA damage in creating de-novo mutations in human offspring: The “post-meiotic oocyte collusion” hypothesis. Reprod. Biomed. Online.

[8] Skakkebaek N.E., Rajpert-De Meyts E., Buck Louis G.M., Toppari J., Andersson A.M., Eisenberg M.L., Jensen T.K., Jørgensen N., Swan S.H., Sapra K.J. (2016). Male reproductive disorders and fertility trends: Influences of environment and genetic susceptibility. Physiol. Rev..

[9] Skakkebaek N.E., Lindhal-Jacobsen R., Levine H., Andersson A.-M., Jørgensen N., Main K.M., Lidegaard O., Priskorn L., Holmboe S.A., Bräuner E.V. (2022). Environmental factors in declining human fertility. Nat. Rev. Endocrinol..

[10] Cannarella R., Gül M., Rambhatla A., Agarwal A. (2023). Temporal decline of sperm concentration: Role of endocrine disruptors. Endocrine.

[11] Centers for Disease Control and Prevention Fourth Report on Human Exposure to Environmental Chemicals, Updated Tables. Atlanta, GA: U.S. Department of Health and Human Services, Centers for Disease Control and Prevention. 2018. https://www.cdc.gov/exposurereport/.

[12] Teuten E.L., Saquing J.M., Knappe D.R.U., Barlaz M.A., Jonsson S., Bjorn A., Rowland S.J., Thompson R.C., Galloway T.S., Yamashita R. (2009). Transport and release of chemicals from plastics to the environment and wildlife. Philos. Trans. R. Soc. Lond. B Biol. Sci..

[13] Warner G.R., Flaws J.A. (2018). Bisphenol A and Phthalates: How environmental chemicals are reshaping toxicology. Toxicol. Sci..

[14] Fujii M., Shinohara N., Lim A., Otake T., Kumagai K., Yanagisawa Y. (2003). A study on emission of phthalate esters from plastic materials using a passive flux sampler. Atmos. Environ..

[15] Lee B.M., Koo H.J. (2007). Hershberger Assay for Antiandrogenic Effects of Phthalates. J. Toxicol. Environ. Health A.

[16] Phthalates—ECHA. https://echa.europa.eu/fr/hot-topics/phthalates.

[17] Aly H.A., Hassan M.H., El-Beshbishy H.A., Alahdal A.M., Osman A.M.M. (2016). Dibutyl phthalate induces oxidative stress and impairs spermatogenesis in adult rats. Toxicol. Ind. Health.

[18] Awny M.M., Al-Mokaddem A.K., Ali B.M. (2021). Mangiferin mitigates di-(2-ethylhexyl) phthalate-induced testicular injury in rats by modulating oxidative stress-mediated signals, inflammatory cascades, apoptotic pathways, and steroidogenesis. Arch. Biochem. Biophys..

[19] Yi W.E.I., Xiang-Liang T., Yu Z., Bin L., Lian-Ju S., Chun-Lan L., Tao L.I.N., Da-Wei H.E., Shang-De W.U., Guang-Hui W.E.I. (2018). DEHP exposure destroys blood-testis barrier (BTB) integrity of immature testes through excessive ROS-mediated autophagy. Genes Dis..

[20] Lyons G. (2000). BISPHENOL A—A known endocrine disruptor. W.W.F. Eur. Toxics Programme Rep..

[21] Goodson A., Summerfield W., Cooper I. (2002). Survey of bisphenol A and bisphenol F in canned foods. Food Addit. Contam..

[22] Vandenberg L.N., Hauser R., Marcus M., Olea N., Welshons W.V. (2007). Human exposure to bisphenol A (BPA). Reprod. Toxicol..

[23] Rochester J.R. (2013). Bisphenol A and human health: A review of the literature. Reprod. Toxicol..

[24] Li D.K., Zhou Z., Miao M., He Y., Wang J., Ferber J., Herrinton L.J., Gao E., Yuan W. (2011). Urine bisphenol-A (BPA) level in relation to semen quality. Fertil. Steril..

[25] Tohei A., Suda S., Taya K., Hashimoto T., Kogo H. (2001). Bisphenol A inhibits testicular functions and increases luteinizing hormone secretion in adult male rats. Exp. Biol. Med..

[26] Liu C., Duan W., Li R., Xu S., Zhang L., Chen C., He M., Lu Y., Wu H., Pi H. (2013). Exposure to bisphenol A disrupts meiotic progression during spermatogenesis in adult rats through estrogen-like activity. Cell Death Dis..

[27] Babu S., Uppu S., Claville M.O., Uppu R.M. (2013). Prooxidant actions of bisphenol A (BPA) phenoxyl radicals: Implications to BPA-related oxidative stress and toxicity. Toxicol. Mech. Methods.

[28] Chitra K.C., Latchoumycandane C., Mathur P.P. (2003). Induction of oxidative stress by bisphenol A in the epididymal sperm of rats. Toxicology.

[29] Bisphenol AF FDS. https://www.sigmaaldrich.com/FR/fr/sds/sial/90477.

[30] Wu D., Huang C.J., Jiao X.F., Ding Z.M., Zhang S.X., Miao Y.L., Huo L.J. (2019). Bisphenol AF compromises blood-testis barrier integrity and sperm quality in mice. Chemosphere.

[31] Brassea-Pérez E., Hernández-Camacho C.J., Labrada-Martagón V., Vázquez-Medina J.P., Gaxiola-Robles R., Zenteno-Savín T. (2022). Oxidative stress induced by phthalates in mammals: State of the art and potential biomarkers. Environ. Res..

[32] Sakaue M., Ohsako S., Ishimura R., Kurosawa S., Kurohmaru M., Hayashi Y., Aoki Y., Yonemoto J., Tohyama C. (2001). Bisphenol-A affects spermatogenesis in the adult rat even at a low dose. J. Occup. Health.

[33] Dobrzyńska M.M. (2016). Phthalates—Widespread occurrence and the effect on male gametes. Part 2. The effects of phthalates on male gametes and on the offspring. Rocz. Panstw. Zakl. Hig..

[34] Meeker J.D., Ehrlich S., Toth T.L., Wright D.L., Calafat A.M., Trisini A.T., Ye X., Hauser R. (2010). Semen quality and sperm DNA damage in relation to urinary bisphenol A among men from an infertility clinic. Reprod. Toxicol..

[35] Damon-Soubeyrand C., Bongiovanni A., Chorfa A., Goubely C., Pirot N., Pardanaud L., Pibouin-Fragner L., Vachias C., Bravard S., Guiton R. (2022). Three-dimensional imaging of vascular development in the mouse epididymis: A prerequisite to better understand the post-testicular immune context of spermatozoa. BioRxiv.

[36] Drevet J.R., Hallak J., Nasr-Esfahani M.H., Aitken R.J. (2022). Reactive Oxygen Species and Their Consequences on the Structure and Function of Mammalian Spermatozoa. Antioxid. Redox Signal.

[37] Aitken R.J., Drevet J.R., Moazamian A., Gharagozloo P. (2022). Male infertility and oxidative stress: A focus on the underlying mechanisms. Antioxidants.

[38] Gharagozloo P., Gutiérrez-Adán A., Champroux A., Noblanc A., Kocer A., Calle A., Perez-Cerezales S., Pericuesta E., Polhemus A., Moazamian A. (2016). A novel antioxidant formulation designed to treat male infertility associated with oxidative stress: Promising preclinical evidence from animal models. Hum. Reprod..

[39] Diniz A., Alves M.G., Candeias E., Duarte A.I., Moreira P.I., Silva B.M., Oliveira P.F., Rato L. (2022). Type 2 Diabetes induces a pro-oxidative environment in rat epididymis by disrupting SIRT1/PGC-1α/SIRT3 pathway. Int. J. Mol. Sci..

[40] Conrad M., Moreno S.G., Sinowatz F., Ursini F., Kölle S., Roveri A., Brielmeier M., Wurst W., Maiorino M., Bornkamm G.W. (2005). The nuclear form of phospholipid hydroperoxide glutathione peroxidase is a protein thiol peroxidase contributing to sperm chromatin stability. Mol. Cell Biol..

[41] Li M.W., Lloyd K.C.K. (2020). DNA fragmentation index (DFI) as a measure of sperm quality and fertility in mice. Sci. Rep..

[42] Vorilhon S., Brugnon F., Kocer A., Dollet S., Bourgne C., Berger M., Janny L., Pereira B., Aitken R.J., Moazamian A. (2018). Accuracy of human sperm DNA oxidation quantification and threshold determination using an 8-OHdG immuno-detection assay. Hum. Reprod..

[43] Luo G., Wei R., Wang S., Wang J. (2017). Paternal bisphenol a diet changes prefrontal cortex proteome and provokes behavioral dysfunction in male offspring. Chemosphere.

[44] Li H., Li J., Qu Z., Qian H., Zhang J., Wang H., Xu X., Liu S. (2020). Intrauterine exposure to low-dose DBP in the mice induces obesity in offspring via suppression of UCP1 mediated ER stress. Sci. Rep..

[45] Zhou D., Wang H., Zhang J., Gao X., Zhao W., Zheng Y. (2010). Di-n-Butyl phthalate (DBP) exposure induces oxidative damage in testes of adult rats. Syst. Biol. Reprod. Med..

[46] Mitsuhashi M., Morimura K., Wanibuchi H., Wanibuchi H., Hayashi S., Kiyota A., Wada S., Nakatani T., Fukushima S. (2004). Di-n-butyl phthalate is toxic to the male reproductive system and its toxicity is enhanced by thioacetamide induced liver injury. J. Toxicol. Pathol..

[47] Zarkovic N. (2003). 4-Hydroxynonenal as a bioactive marker of pathophysiological processes. Mol. Aspects Med..

[48] Prados J., Stenz L., Somm E., Stouder C., Dayer A., Paoloni-Giacobino A. (2015). Prenatal exposure to DEHP affects spermatogenesis and sperm DNA methylation in a strain-dependent manner. PLoS ONE.

[49] Mokra K., Woźniak K., Bukowska B., Sicińska P., Michałowicz J. (2018). Low-concentration exposure to BPA, BPF and BPAF induces oxidative DNA bases lesions in human peripheral blood mononuclear cells. Chemosphere.

[50] Ferreira R., Amaral C., Correia-da-Silva G., Almada M., Borges M., Cunha S.C., Fernandes J.O., Teixeira N. (2022). Bisphenols A, F, S and AF trigger apoptosis and/or endoplasmic reticulum stress in human endometrial stromal cells. Toxicology.

[51] Liang S., Yin L., Shengyang-Yu K., Hofmann M.C., Yu X. (2017). High-content analysis provides mechanistic insights into the testicular toxicity of bisphenol A and selected analogues in mouse spermatogonial cells. Toxicol. Sci..

[52] Harnett K.G., Chin A., Schuh S.M. (2021). BPA and BPA alternatives BPS, BPAF, and TMBPF, induce cytotoxicity and apoptosis in rat and human stem cells. Ecotoxicol. Environ. Saf..

[53] Aitken R.J., Drevet J.R. (2020). The importance of oxidative stress in determining the functionality of mammalian spermatozoa: A two-edged sword. Antioxidants.

[54] Drevet J.R., Aitken R.J. (2020). Oxidation of sperm nucleus in mammals: A physiological necessity to some extent with adverse impacts on oocyte and offspring. Antioxidants.

[55] Chabory E., Damon C., Lenoir A., Kaulselmann G., Kern H., Zevnik B., Garrel C., Saez F., Cadet R., Henry-Berger J. (2009). Epididymis seleno-independent glutathione peroxidase 5 maintains sperm DNA integrity in mice. J. Clin. Investig..

[56] Wood M.L., Esteve A., Morningstar M.L., Kuziemko G.M., Essigmann J.M. (1992). Genetic effects of oxidative DNA damage: Comparative mutagenesis of 7,8-dihydro-8-oxoguanine and 7,8-dihydro-8-oxoadenine in *Escherichia coli*. Nucleic Acids Res..

